# Constructing childhood depression: a qualitative study with international experts in child psychiatry

**DOI:** 10.1007/s00787-023-02270-0

**Published:** 2023-08-30

**Authors:** Alexis Rayapoullé, Marine de Chassey, Laelia Benoit, Christine Hassler, Bruno Falissard

**Affiliations:** 1 Centre de recherche en épidémiologie et santé des populations (CESP), Inserm U1018, Université Paris Saclay, Villejuif, France; 2 Assistance publique–Hôpitaux de Paris (AP-HP), Paris, France; 3 Child Study Center, Yale University, New Haven, CT USA

**Keywords:** Depression, Qualitative research, Child psychiatry, Mental disorders, Epistemology

## Abstract

After decades of controversy, the concept of childhood depression now seems to be part of standard medical knowledge. Yet the form and content of this nosological entity, like many psychiatric diseases, is continuously shaped by the scientific, clinical, and political communities involved in child psychiatry. In this qualitative study, we explored how the concept of childhood depression is constructed in early twenty-first century child psychiatry. We conducted a series of 18 interviews with practising child psychiatrists, international experts in the field, and interpreted them with thematic analysis informed by discourse analysis. We identified five overarching discourse themes across interviews, relating to the definition of depression, the diagnostic process, the causes of this condition, the therapeutic strategy, and the scientific role of child psychiatry. Most participants agreed that childhood depression was a mental disorder where irritability prevailed, heavily influenced by psychosocial factors, and for which psychotherapy was the ideal treatment. However, subtle points of dissent also surfaced: whether depression is primarily a mood state or psychological suffering, whether categories or dimensions are more suitable to make the diagnosis, and whether there is a genetic predisposition were some of the most controversial topics. Theoretical considerations regarding childhood depression may have significant scientific, moral, and socio-political implications beyond child psychiatry and should be addressed appropriately.

**Supplementary Information:**

The online version contains supplementary material available at 10.1007/s00787-023-02270-0.

## Introduction

In 2021, the World Health Organization (WHO) considered depression to be a common illness all over the world, affecting 3.8% of the global population (280 million people in 2019) [1]. As such, the WHO qualified depression as the “*leading cause of disability worldwide*.” Like all nosological entities, particularly psychiatric ones, the concept of depression has been described, defined, and used in many ways in the history of medicine. A popular belief is that words have true meanings attached to fixed objects and phenomena of reality. Yet, reality does not speak for itself; we humans attempt to arrange our perceptions of this reality with abstract words [2]. Scientific concepts are no exception: they have not designated and will not always designate the same phenomena, nor have they occupied or will they always occupy the same place within our body of knowledge. Our perspective in the following falls precisely within this constructivist approach: all scientific consensus is temporary, and the form and content of concepts are continuously shaped by the people using them [3].

The pathologisation of out-of-proportion fear and sadness goes back to Hippocrates with the concept of melancholia, although its properties (delusions, risk of mania and dementia when left untreated) could make it as much a precursor of modern depression as of schizophrenia and bipolar disorder [4]. In the early twentieth century, ‘depression’ overtook ‘melancholia’ and was conceived, in the psychoanalytic tradition, as a defence mechanism against anxiety neuroses [5]. In the 3rd edition of the Diagnostic and Statistical Manual (DSM-3) of the American Psychiatric Association (APA), all references to psychoanalysis were abandoned in favour of a claimed atheoretical classification of mental disorders. The concept of major depressive episode (MDE) entered the taxonomy as part of major affective disorders, markedly distinct from anxiety disorders [6]. A syndromic approach based on clinical symptoms was preferred to etiological speculations, in which dysphoric mood and/or loss of interest or pleasure in all or almost all usual activities and pastimes were—and still are—the chief characteristics. This clinical shift in the definition of depression was decisive to the current pre-eminence of this disorder among psychiatric illnesses. The recent epidemiological outburst of depressive conditions could be explained by an ever-increasing depressogenic environment to which human beings are maladapted [7], but some argue it is better explained by these changes in the criteria than changes in actual rates of depression [8].

Depression in children became conceivable in the second half of the twentieth century: another significant conceptual shift. René Spitz and Katherine Wolf coined the term “*anaclitic depression*” in 1946 to describe infants separated from their primary caregiver, whose behaviour was similar to adults diagnosed with depression [9]. Weinberg et al. seem to have been the first, in 1973, to describe depression in children aged 6–12 years, dominated by irritability and hyperactivity [10]. Before the 1970s, in Western psychiatry, most psychiatrists seemed to consider severe depression as impossible in children based on theoretical assumptions [11]. The epistemological shift introduced by the DSM-3 later created some space for children-specific criteria of MDE. In the latest version of the classification (DSM-5-TR), major depressive disorder (MDD) is still very similar to the clinical picture formerly labelled MDE, with the specification that in children and adolescents, depressed mood can be irritable mood [12]. Due to concerns about the potential overdiagnosis of bipolar disorders in children, another depressive disorder was created in the DSM-5 and restricted to children 6–18 years: disruptive mood dysregulation disorder (DMDD), in which temper outbursts, irritability, and anger are also the dominant characteristics [12, 13]. All in all, persistent and pervasive sadness of mood, loss of interest in activities previously enjoyable, boredom, tearfulness, difficulty concentrating, feeling worthless, feeling fatigued, changes in weight and appetite, school-related difficulties (academic decline, school refusal), temper tantrums, and irritability make, at the present day, the typical childhood depressive syndrome in the biomedical scientific literature [14]. Based on these criteria, the prevalence of prepubertal depression is estimated at 1–2%, an onset before 9 years old appears highly improbable (although the incidence seems to increase and the age of onset to decrease with time), and an episode lasts on average 7–9 months with a remission up to 90% at 1.5–2 years, but a recurrence of the disorder up to 40% at 2 years and 70% at 5 years [15].

In short, both concepts of depression and childhood depression seem to have been relatively unstable and changing over the past decades. It is an excellent opportunity for us to study the ‘science in action’ as sociologist Bruno Latour once wrote [16]. Manuals and scientific articles are worthy study materials to explore the history of ideas and research programmes, but interviews with prominent researchers and clinicians is a better way to identify the present doubts, motivations, and certainties that will make the future of psychiatry, before the ‘black box’ closes and knowledge is stabilised. How do contemporaneous experts of child psychiatry use, define, and understand the concept of childhood depression? And what are the implications of such instability? Reflecting on the pertinence of psychiatric nosological categories was at the core of the atheoretical ambition promoted by the DSM-3 authors, who viewed their classification as temporary hypotheses to be revised. In this study, we propose to carry on this endeavour with the use of qualitative methods of investigation. Our goal is to assess the rationale underlying the use of childhood depression as a mental disorder category, to help guide its refinements and future developments.

## Materials and methods

Our study consists of an analysis of interviews with experts in the field of child mental health. The research team was made of five members of a research unit with expertise in the analysis of social and cultural determinants of mental health, three of whom are psychiatrists. The core analysis and writing were performed by physicians trained in public health, social sciences, and philosophy. Thus, the background of the team led to an international design with a constructivist perspective. The impact of culture, epistemology, and professional practices was a specific focus for this research, hence the use of thematic analysis informed by discourse analysis. Debriefing was held on a regular basis with an outside group of researchers in social sciences (Grounded Lab) to ensure good reflexivity on our part, in addition to the methodological rigour we applied ourselves.

Using contacts provided by the International Association for Child and Adolescent Psychiatry and Allied Professions (IACAPAP) [17], two researchers (BF, MDC) selected 18 child psychiatrists in an attempt to have the broadest spectrum of location, age, gender, and epistemology (which aspects of the biopsychosocial framework of their discipline seemed the most meaningful to them: biology, social sciences, psychology) based on their clinical, scientific, and academic activities (including research topics and preferential activities in professional societies such as the IACAPAP). With this method, we tried and collected as many different perspectives as possible from socially and scientifically recognised experts. All contacted experts agreed to participate.

The interviews were conducted in 2020 by MDC alone by videoconference (except for one, face to face) and lasted 30–65 min, following a semi-structured guide. Sensitising questions addressed the following topics: the existence and definition of childhood depression, its differences with adult and adolescent depression, the pertinence of the DSM criteria to define the disorder, the clinical signs leading to a diagnosis of childhood depression, the usefulness of scales for practice and/or research, the meaning of somatic complaints, their potential encounter with suicidal children, their preferred treatments, the perceived risk factors and comorbidities, the truth and impact of genetic predisposition, and the relationship between child depression and subsequent psychopathology (see Supplementary materials). All the interviews were recorded with the participants’ consent and transcribed by MDC. In the end, the sample included 6 women and 13 men; their ages ranged from 45 to 78, with a mean of 58.2 years (SD = 10.4). Seven were conducted in French, MDC’s mother tongue, and 11 were conducted in English (among which 6 were with non-native speakers). Quotes that were originally in French were translated into English and are marked by an asterisk (*). The participants practised in Europe (*n* = 7) (two in France, one in Iceland, UK, Croatia, Spain, and Germany), North America (*n* = 4) (two in the USA, one in Canada and Mexico), Africa (*n* = 3) (Tunisia, Morocco, Egypt), Asia (*n* = 2) (Singapore, Japan), South America (*n* = 1) (Chile), and Oceania (*n* = 1) (Australia). It should be specified that not all of them were born in or were citizens of the country they practised in, nor were they necessarily trained there. All of them produced academic work and had clinical activities.

Two researchers (AR, CH) analysed the transcripts in 2022–2023, using a qualitative analysis software (NVivo) to conduct a thematic analysis informed by discourse analysis [18]. A primary coding of each interview was performed (following the chronological order) and revealed that data sufficiency had been reached after interview #15. After confirming that interviews #16 to #18 appeared to overlap with earlier ones, the research team decided not to resume recruitment and carry on with the transversal analysis. AR and CH then independently formulated themes and labels following an inductive process, a constructivist epistemology and an interpretative framework. AR and CH then confronted their findings and agreed upon a tree map of nodes for analysis. Both researchers coded three interview transcripts separately to triangulate the themes induced from the data and ensure that the attribution of quotes to nodes was sufficiently similar. This process led to a minor revision of the tree map. AR coded the rest of the interviews and performed the transversal analysis, i.e. the main ideas brought up by participants, if they were unanimous or divided, if they could be linked to some sociodemographic elements, and if they were related to and/or redundant with other themes and subthemes. The entire research team discussed the overall themes, and all co-authors contributed to the final manuscript.

## Results

**Fig. 1:**
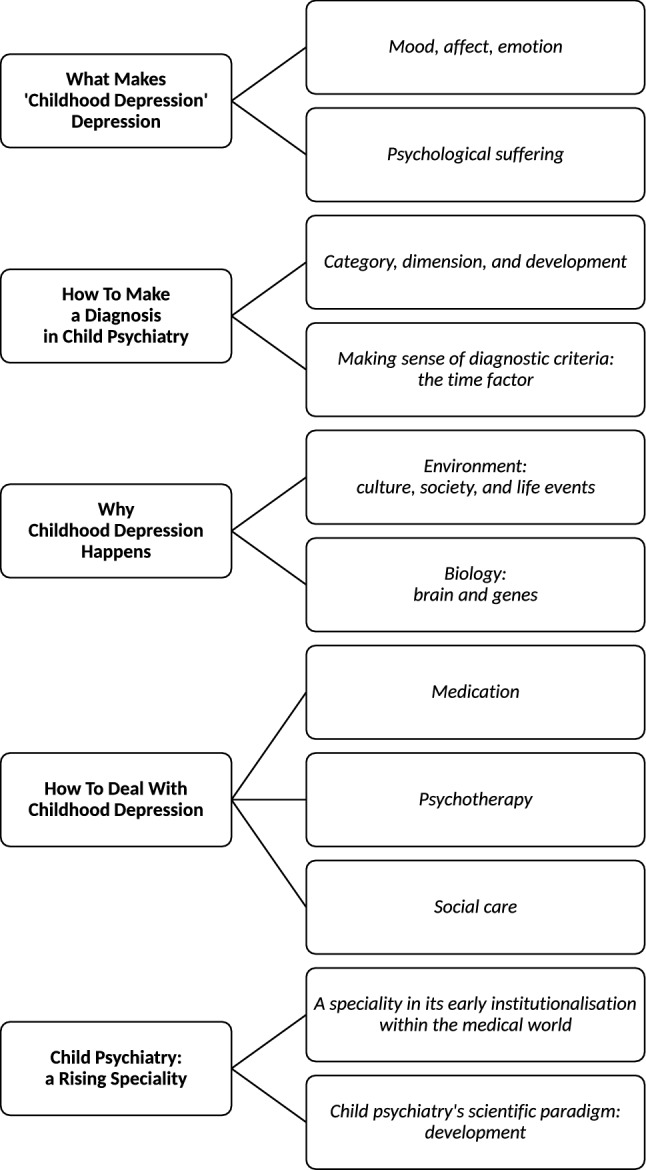
Final tree map of nodes: 5 themes (left) and 11 subthemes (right)

We will present the results in five themes and their subthemes (see Fig. 1): (1) what makes ‘childhood depression’ depression (mood, affect, emotion/psychological suffering); (2) how to make a diagnosis in child psychiatry (category, dimension, and development/the time factor); (3) why childhood depression happens (environment/biology); (4) how to deal with childhood depression (medication/psychotherapy/social care); and (5) child psychiatry: a rising speciality (institutionalisation/scientific paradigm).

### What makes ‘childhood depression’ depression

Our interviewees almost unanimously considered childhood depression as a mental disorder affecting the mood. But whether the mood itself was the core of the definition or a consequence of a particular psychological state seemed to vary among participants.

#### Mood, affect, and emotion

Many participants considered the emotions felt by children as the central item to the definition of a depressive episode. They agreed that “*low mood*” (P5, P6, P7, P10, P14), “*depressed mood*” (P7, P11, P13, P18), or “*sadness*” (P1, P2, P3, P4, P5, P6, P7, P8, P9, P12, P15, P16) were central definitional items in adult depression, but also agreed to claim that it was largely optional in the case of children, with irritability and anger being the primary affects. One participant even said that sadness and low mood were beyond optional: they are arguments against childhood depression.

When I witness an obvious case of sadness with some psychomotor retardation and suicidal ideation, I am suspicious. It’s a child who borrows from the adult depressive repertoire. I am against diagnosing a child that easily. (P15*)

For this participant, a child who displays obvious sadness instead of irritability might be mimicking adults surrounding them, meaning that they are might appear depressed but are not authentically depressed.

What usually distinguishes mood from emotion is its duration and stability, contrary to the transience and instantaneity of the latter [19]. In adults, ‘depressed mood’ or ‘low mood’ is considered a state of profound and permanent sadness with loss of interest and pleasure [20]. But interviewees also insisted on the lability of mood in children in general, including those diagnosed with depression.

They might be very very depressed and the next minute we might get them involved in doing something and then they turn back to being depressed. They are very ‘here and now’ creatures. (P6)

These views negate the fundamental link between sadness/anhedonia and depression as defined in adults. Some participants were therefore led to hold childhood depression as a specific paediatric disease, qualitatively distinct from adult depression. For instance, Participant 9 developed three arguments in favour of childhood depression being a specific and separate disease: a balanced sex ratio up until adolescence, the inexistence of psychotic forms of depression before puberty, and a poor response to pharmaceutical treatments. With these viewpoints, the link between childhood depression and depression becomes blurry at best, if not meaningless, thus questioning if childhood depression should be called ‘depression’ at all. One participant did draw a similar conclusion:

Yes, a child will be abused. Yes, a child will not do well. Yes, they will be agitated instead of sad. All that is true. But is it depression? (P1*)

This participant argued that individuals organise their mental life in accordance with concepts they have acquired in the past. In adults, it would be structured around the notion of ‘loss’ and result in depressive disorders (major depression, dysthymia, etc.). In children, the psychological reaction (here because of past trauma, see theme #3 ‘why childhood depression happens’) would be different because the structure of their mind is not the same, resulting in a different mood state and behaviour (irritability). With this perspective, depression would not be a clinical picture, but a precise psychopathological reaction. Participant 1 claimed he was therefore “*embarrassed*” to call these psychological phenomena in children ‘depression’ if they were “*phenomenologically different*.”

#### Psychological suffering

Participant 1 was the only psychiatrist showing scepticism about the similar nature of psychological mechanisms between depressed adults and children. Most participants considered that depression existed in children and adults, leaving the clinical variations only to how depression is expressed.

Although participants agreed on the clinical picture, several distinguished a definition of depression based on mood and emotions from a psychological definition based on the subjective interpretation and meaning one person attributes to their experiences. As such, Participant 15 taught his students to separate the “*clinical discussion*” (a syndromic approach based on the mood) and the “*psychopathological discussion*” to address the possible meaning and function of depression in the child’s life.

Let’s think of this more broadly than just as a disease condition, let’s think of this as a feature of the way life seems to this person right now. (P12)

These participants mainly defined depression as an intellectual experience of suffering. Participant 12 avoided labelling children with the diagnosis of ‘depression’ and would instead use what he termed an “*empathic diagnosis*”: a statement co-constructed with the child about what they feel and think of themselves and the world, such as “*you feel like there’s nothing you can do to change your life*,” “*you feel like your life is not good*,” “*you feel that nobody understands what’s going on*.” Other participants phrased similar statements capturing low self-esteem and feelings of injustice (P8, P9, P16). Participants considered that the emotional and behavioural expression of depression varied following the child’s age due to young children’s presumed limited verbalisation capacities.

It’s a mood disorder and there are some differences because it depends on the age of the child: they cannot express in words what they are living or what they are feeling so maybe, it is more of a behaviour thing than in other ages. (P13)

Aware of this variability in the presentation of depression in children, the participants insisted on the importance of a developmental perspective on psychiatric disorders.

### How to make a diagnosis in child psychiatry

Diagnosis is both a category and a process [21]. This section focuses on diagnosis as a process, i.e. how participants applied the label of childhood depression.

#### Category, dimension, and development

Most of our participants heavily criticised the diagnostic criteria of major depressive disorder as established in the DSM-5. For some participants, the problem was its categorical approach; they felt a dimensional one would be more suitable, i.e. that there is no qualitative difference between normal and disordered, only a difference in intensity of symptoms (see Discussion). In a way, they reconnected with the psychoanalytic view of mental disorders, in which depression and neuroses in general were continuous dimensions. They did not, however, necessarily re-engage with the content of these theories.

Childhood depression would be, in my opinion, more dimensional, let’s say, than a clear-cut nosographic picture as we see in adults. (P15*)

Most participants, however, considered the categorical approach to mental disorders adequate for child psychiatry. The DSM-5 appeared to be a disappointing clinical tool because of the content of the categories, not because of the approach itself. The mention of irritable mood potentially replacing depressive mood is, in this perspective, a step forward in the conception of a child-specific clinical picture. In that respect, they wished for refined categories matching children’s developmental stages.

You have to admit that the criteria are beginning to show some developmental differentiation. But for now, the risk is that one might use the adult criteria without knowing these fundamental developmental notions. (P16*)

Finally, one participant made an explicit difference between the lay sense of the word ‘depression’, perhaps blurrier and more continuous with normal states, and the clear-cut syndromic approach in the medical sense.

[Depression is] the term that now everyone knows because they didn’t succeed at an exam or they didn’t succeed at the sports game; they are ‘depressed,’ but it’s not like the clinical depression. (P5)

#### Making sense of diagnostic criteria: the time factor

Participants massively advocated for what we could call a longitudinal diagnostic process over a transversal one. They considered that the chief alert was not so much a state, like a bored or an irritable child, but a rupture in time: a previously energetic and vivacious child who would lose interest in life. “*Change*” was the keyword to describe this diagnostic process (P7, P8, P9, P15, P16, P18).

You have to look for a change compared to the previous state because some children have always had… Parents tell me, ‘He has always had this look. He has never been a very social or funny kid.’ (P8*)

Incorporating a time factor in the diagnostic process gives parents a particular role that clinicians alone cannot fill. Many participants considered the parents as keen observers of their child, almost like whistleblowers (P2, P3, P4, P5, P6, P8, P12, P15, P19).

First of all, I think you have to trust parents when they come in and they say, ‘There’s something wrong with my child.’ That’s the starting point. Because parents are, for the most part, very good observers of their children. (P3)

### Why childhood depression happens

This section will expose all the elements in our participants’ discourses regarding the various factors leading to childhood depression. No participant claimed that there was one unique and identifiable cause of this condition. All participants mentioned many underlying, triggering, and aggravating factors, never sufficient nor necessary by themselves, and integrated those within a biopsychosocial-like model of depression. Yet, they did not all give the same weight to the bio-, psycho-, and social components in their understanding of this phenomenon. In particular, genetics seemed to be a point of controversy.

#### Environment: culture, society, and life events

Many words used by our interviewees positioned the causes of childhood depression as external to the child: “*environmental*,” “*psychosocial*,” “*socio-cultural aspects*,” “*family structures*,” “*social stability*,” “*trauma*,” “*loss*,” “*reaction to the world*,” “*situational*”, and “*life story*.”* One participant even used the term “*psychopathology of the environment*” to emphasise how much the social environment matters to a child’s mental health.

We sorted these environmental factors into three categories. The first refers to structural social factors: poverty, social adversity, socio-cultural challenges, and academic pressure (P2, P3, P13). In particular, participants from North African countries (Tunisia and Morocco) stressed that school systems in their countries caused significant psychological harm to young children. One participant even referred to “*school terrorism*” to describe the violence schools exerted on children.

An important risk factor: school pressure is a sort of school terrorism exerted on children.*

Psychiatrists from North African countries stressed that undue academic pressure and interpersonal challenges such as bullying could induce suicidal ideations in children as young as 7 years old, and that suicide attempts in children aged 6–12 years had increased in the last five years. One participant mentioned that the recent political history in North African countries has led to considering suicide as empowering, because it can be seen as heroic and socially desirable, counteracting feelings of worthlessness often associated with such mental states.

Death is seen as a solution. It is glamourised in this very particular economic context. In Tunisia, we have been since 2011 in an era we qualify as ‘post-revolutionary.’ Many adults have committed suicide, thankfully fewer now. But every time there is a problem, or someone sees an obstacle along the way, they threaten to set themselves on fire in the street.*

The second type of external factors we have identified is significant life traumas (P1, P4, P5, P6, P8, P13, P14, P16, P17). These included physical, emotional, and sexual abuse, neglect, domestic violence, and bullying. According to participants, such painful events could throw off balance a child’s mental stability. But our interviewees also framed other life events as potentially traumatising: bereavement, parental divorce, problematic family interactions (e.g. parental depression), and academic struggle were considered triggers for child depression.

Of course life events, we all know, would have an important role to play. More and more we are seeing adverse childhood experiences, childhood trauma, traumatic events for attachment relationships, current relational issues, break-up in relationships whether it’s family relationships or whether it’s, you know, boyfriend-girlfriend relationships: any break or loss of any kind would all be considered as factors. (P17).

The third environmental factor we identified was the social response to other disorders. Participants framed the environment’s reaction to comorbidities—namely, stigma affecting the child’s self-esteem—as a risk factor for depression. Participants described chronic somatic diseases as negatively impacting family bonds.

With these children, there is a chronic problem coming from the—we say ‘physical’, although I don’t like separating [from mental]—disease, and it is always pretty tough for families. It’s a public hospital, they are poor people with a long-term [comorbid] medical problem, and these people have a lot of difficulties going through this. So depression is almost a logical consequence. (P2*)

Anxiety, attention-deficit hyperactivity disorder (ADHD), learning disabilities, developmental language disorders, and obsessive–compulsive disorders were described as distressful in a maladapted school system once children became aware of their academic difficulties. Children with autism, whose interactional challenges are not considered in school settings, were said to experience isolation. As these children had several issues entangled with one another, participants struggled to address them separately.

What we do have is a combination of things, so we have plenty of children that have ADHD and depressive features or some conduct disorders that have some depressive features, but it’s very rare that we get just a depressed case. (P4)

Some participants mentioned the conceptual difficulties of distinguishing ‘pure’ or ‘isolated’ depression (without any external cause) from a depression triggered by a psychosocial experience, thus highlighting how debates on causes and definitions of disorders are fundamentally linked (P2, P5). But others did not seem to consider depression as less ‘real’ when it was solely induced by psychosocial events (P7, P11).

Children from age 10 to 12 come and say, ‘I’m depressed,’ but don’t have emotional support in their families; it’s not like a real depression. (P5)

It is definitely a comorbid disorder, and the other disorder is the major disorder. So obviously, major depression is a common comorbid disorder. (P11)

These quotes show how participants can have differing views on what it takes to have a true depression. Participant 5 considered that lack of family support (a legitimate psychosocial cause for many other participants) was not enough to cause a “*real depression*,” whilst for Participant 11, the negative psychosocial impact of other disorder was compatible with depression as a rightful comorbid disorder, not just as a figure of speech.

All participants mentioned anxiety as closely linked to depression. Our interviewees expressed various opinions: anxiety and depression as distinct and exclusive disorders, as separate but frequently co-occurring in a common anxiodepressive syndrome, and anxiety as a cause of depression through emotional exhaustion. Several of them interpreted somatic complaints as anxiety consequences rather than depressive equivalents (P9, P11, P13, P15).

The somatic complaints, I wouldn’t immediately associate them with childhood depression. […] I see them more in relationship to anxiety disorders. Obviously, anxiety disorders can also be associated with, so it could be comorbid depression. But the somatic complaints would most likely tend to be associated more with the anxiety disorder than the depression itself. (P11)

In adults, the differential diagnosis between depression and anxiety is a well-known challenge [22]. But more specific to children, and due to irritability being the main agreed-upon symptom of childhood depression, participants also mentioned the challenge of differential diagnosis between depression and ADHD. Are they exclusive? Does ADHD cause depression? And if so, how do we diagnose the latter?

Many children have these acting-out reactions because of the irritability, and we have to make assessment whether it’s a behavioural disorder or a depression. But children with depression usually feel guilty when they act out aggressively. So that’s how we distinguish depression from ADHD. (P5)

Conjunctions of adverse life events and social hardship remained at the forefront of participants’ understanding of children’s mental health. This led several participants to consider childhood depression as a fundamentally reactive disorder, meaning that environmental factors have more impact on childhood depression than on adult depression.

#### Biology: brain and genes

Participants also made use of a biomedical vocabulary when describing childhood depression: “*neurobiological*,” “*genetic predisposition*” or “*load*,” “*genetically based mood disorder*,” “*family history*,” “*biological determination*,” “*neurohormonal*,” and “*familial genetic structure*.”* Almost all interviewees acknowledged biological causes of depression, while still downplaying their relevance (P1, P2, P4, P5, P6, P8, P10, P11, P12, P13, P14, P17, P18). Most of them considered that the biomedical model of depression was epistemologically weak.

Genetics is a small component; the bigger one is the psychological factors. (P10)

A minority still held dominant biological explanations. One participant, prompted by his work on hormonal causes of childhood depression, believed there might be different subtypes of depressive disorders with different aetiologies (P11). Another participant considered biology as the leading cause of depression and environment as contingent (P3).

There are environmental factors that play a role in mood disorders. I suspect they may not account for a huge portion of the aetiology, but they certainly can count for a huge portion of the aggravating factor. (P3)

While no participant rejected the idea of an environmental influence on the onset of depressive disorders, some entirely denied genetic factors any explanatory power for childhood depression (P7, P9, P15, P18).

I don’t believe it [genetic predisposition to childhood depression] one bit. I find it totally absurd. Data absolutely do not go this way. We are just saying that psychiatric conditions have something to do with brain development, which we already know. So, I am very cautious. (P9*)

In sum, all the participants agreed that childhood depression is more rooted in environmental issues and adverse childhood experiences than adult depression, whether they framed depression more as a biomedical or a psychosocial condition. Disagreements revolved around the status of biology and genes as significant causal factors of childhood depression. Whereas this argument does not seem to have much consequences in terms of treatment strategy (see theme #4: how to deal with childhood depression), it has a lot of consequences on how parents are perceived by clinicians. They can be made powerless or responsible according to how we attribute the cause of the disorder.

It’s not parents who cause it. And I think we need to stay away from, when we do diagnoses and we talk about treatments, we need to be very careful to not blame anybody, because I think that’s just not very helpful in the process of treatment and recovery. (P3)

[I say to the parents] “*they’re hurt, look, it’s normal! They hear you yell at each other, they sometimes hear shouting and are afraid for themselves*”. […] Just saying “*stop messing around*” [to the parents,] they understand 3 out of 4 times. (P1*)

### How to deal with childhood depression

Discourses on the treatment of childhood depression were the most homogenous among all participants.

#### Medication

All participants considered medication as a symptomatic treatment, never sufficient on its own, and unable to address the cause of depression. Two participants (P3, P17) mentioned using medication as a first-line treatment alongside psychotherapy. But most participants (P4, P6, P7, P10, P11, P14, P15, P16) referred to medication as a second-line option after failure of psychotherapy. Fluoxetine was by far the preferred medication, but sertraline was also brought up on a regular basis. Escitalopram, aripiprazole, and tricyclics were occasionally mentioned to handle specific situations.

After three or four weeks [of psychotherapy], we would start with an antidepressant. For children, basically, almost always fluoxetine. (P11)

Participants who did not systematically prescribe medication provided insight into what would make them consider pharmaceutical treatment. Most stated that severe depression with suicidal ideation or behaviour would lead them to immediately prescribe medication (P2, P3, P4, P6, P8, P9, P13, P14, P18). Three participants used the anxiolytic effects of SSRIs to treat impairing co-occurring anxiety symptoms (P1, P14, P15). One participant initiated an antidepressant medication when the clinical assessment concluded that the child was at risk for a severe episode: if the depression had started long ago, if there was a family history of depressive disorders, and if the symptoms were numerous (P12).

For all the depression disorders, the important is psychotherapy, and this psychotherapy must involve the family and also the school in most of the times. But for severe, it’s also important to include antidepressants. (P13)

The FDA authorisation for fluoxetine to treat childhood depression is based on evidence for children above 8 years. When asked about it, four participants excluded prescribing any medication for children under 8 years old, either to avoid using a psychoactive substance on a yet developing brain without enough evidence, or simply because they found it unnecessary (P2, P4, P6, P8).

MDC*: Recommendations speak of fluoxetine above 8 years old. Below 8, do you sometimes prescribe? P8*: Never. I think there is not enough evidence or clinical trials. It is still a developing brain.

An interesting feature of the participants’ discourse about treatment was how family preferences were essential in deciding whether to prescribe medicine (P5, P9, P14, P19). Participants stressed the importance of a good therapeutic alliance and considered parents’ views on pharmaceutical treatment as rooted in cultural aspects. One participant described parents as afraid of the effect of psychotropic medication on their child’s brain development (P14). Elsewhere, depriving a child of a drug was like prolonging their suffering and wasting the time needed to grow up healthy (P18).

Some parents get very alarmed because the child feels very tired and drowsy because whenever they are given psychotropic medicine, at least in [my country], they think it’s working on the brain and the brain is very important for studying. (P14)

There are many cultural things in this. What a family [of my nationality] would like is categorically refused by a family in [my country of practice]. Actually, and in contrast, families [here] want the pharmaceutical treatment because they consider that depriving the child of treatment is to make them suffer for no reason, and to waste their precious development time. Whereas in [my country of origin], it is the opposite. [There], we consider that we have to avoid medication at any cost, that it is toxic for the development of the nervous system, and delay and avoid as much as possible. So, in my practice, I do like [people here] because I am [here]. (P18*)

Lastly, some participants thought that fluoxetine was probably inefficient. This, however, did not prevent them from prescribing medication in specific contexts. They seemed to consider drug therapy as useless but not deleterious.

Patients with childhood depression often do not respond to drug therapy. (P7)

#### Psychotherapy

Contrary to medication, which was considered a symptomatic treatment by all participants and often limited to the most urgent and severe cases, psychotherapy was unanimously designated as the gold-standard treatment of childhood depression.

The technique of choice, however, varied greatly among interviewees and was often very eclectic within interviews. Cognitive behavioural therapy (CBT) was the most cited psychotherapeutic approach (P3, P4, P5, P6, P7, P8, P10, P11, P12, P13, P14, P15, P16, P17) and the preferred one for these psychiatrists, except for two participants. The second most cited approach was family therapy (P5, P8, P11, P13, P14, P18). Psychodynamic therapy was promoted by three participants (P6, P8, P14), and could be their first-line choice in specific situations (for children under 6 years old or with a history of trauma and abuse). Group therapy was also mentioned as producing good results (P5, P19), although sometimes prompted by the material impossibility of performing individual therapy. Other types of therapies were art therapy (P8, P11), psychoeducation (P7, P11), applied behaviour analysis (P7), recreational therapy (P10), animal-assisted therapy (P10), and talking therapy (P11, P12).

Two participants (P1, P9) stepped out of this discussion about the best psychotherapeutic approaches because they were not convinced that the technique mattered; or if it did, much less than the quality of the human relationship between a therapist and their patient.

When a child sees a shrink, it’s not because the professional is labelled ‘psychoanalyst’ or ‘behaviourist’ that it’s in any way meaningful to them. The child comes to see an adult who cares about them, who asks them questions, and who wants to help them. In the end, even an ignorant can help a depressed child. (P9*)

Participants’ discourses revealed what they considered to be the ideal care for depressed children. But most interviewees mentioned the gap between theory and practice, as the economic system in which they work constrains the quality of care: a lack of trained psychotherapists, too few practitioners to maintain an optimal follow-up frequency, not enough consultation time (P1, P2, P3, P5, P6, P7, P13, P15, P16, P18). Such economic constraints led them to rely on sub-optimal psychotherapy techniques, interspaced follow-ups, and in the end, to increase the use of medication.

Unfortunately, most cases do not have immediate access to the type of psychological therapies we want. So the question is: should we initiate medication even if the guidelines clearly say that medication should be used in combination with psychological treatments? (P16*)

In sum, all participants claimed psychotherapy was the first-line and ideal treatment. CBT appeared widely favoured, but many interviewees had an eclectic approach, motivated either by theoretical pluralism or by practical constraints. Family therapy, for one, seemed relatively popular.

#### Social care

Some participants called for an action that, in our analysis, goes beyond psychological care and could be referred to as social care (P2, P5, P7, P9, P11, P15). Their primary motivation was considering the child’s environment a significant lever to alleviate their suffering.

When the family was thought to be dysfunctional at worst and overwhelmed at best, some interviewees suggested groups of discussion, information, and guidance with parents. One participant even saw hospitalisation as a form of exfiltration from an inadequate domestic environment (P11).

We remove the kids from home for one, which is a major intervention. Then we provide the kids with a daily structure which they commonly did not have prior to coming into the unit. (P11)

If school was perceived as the main problematic environment for the child, like cases of bullying or tremendous academic pressure, participants advocated working with teachers and suggesting school arrangements (P7, P9, P15). One participant involved social welfare services (P5) for a more comprehensive approach. For most of these participants, these forms of social care were complementary, perhaps secondary, to the aforementioned pharmaceutical and psychological treatments. One participant however, made it clear that improving the environment was the primary treatment for childhood depression.

When choosing treatment, I mainly adjust environment for childhood depression. (P7)

In sum, although most participants recognised the importance of environmental factors as triggers or aggravators of childhood depression, only a few claimed to act directly upon this environment. Perhaps they believed it was beyond their mission as physicians, or out of their control, or that these environmental factors were past events and not ongoing situations.

### Child psychiatry: a rising speciality

This last result section highlights how the participants viewed their professional role and scientific knowledge in a larger health-care ecosystem, beyond childhood depression.

#### A speciality in its early institutionalisation within the medical world

Participants from different geographic areas shared how their medical education had been based on adult psychiatry, and how child and adolescent psychiatry had only recently become an independent speciality (P2, P8, P13, P18).

We have just started, two years ago, a training programme for residents. Child psychiatry becomes, for residents, a medical speciality in its own right, ‘independent’ if I may say so, which means we no longer depend on adult psychiatry to train residents. (P18*)

#### Child psychiatry’s scientific paradigm: development

The notion of development was already explored to analyse its impact on how our participants theorise and classify mental disorders. It surfaced again here as a way to unify child psychiatry with adult psychiatry while maintaining its specificity. Participant 16, for instance, explicitly promoted the developmental paradigm as a key research programme for academic child psychiatry.

I think we have to push more as an academic child psychiatry unit to really show the differences relating to development. (P16*)

With these developmental goggles, depression is thought to be a continuous disease—despite various expressions—between children, adolescent, and adults. Yet, scrutiny of some participants’ discourse showed that this continuity was often considered due to a persistence of the psychosocial causes (a deleterious environment) rather than an inner vulnerability to depressive disorders (P5, P6).

There is a continuation, particularly if the psychosocial situation is continuous, such as parental discord, abusive family, or domestic violence. If the psychopathology of the environment continues, I think you don’t have much chance not to continue with your psychopathology. (P6)

Some participants even doubted that there was such continuity between child depression and adult depression (P1, P10, P15).

MDC*: Do you think that depression in a child can lead to a psychological vulnerability at adult age? P15*: Not particularly. I even think it can be maturing. I here go back to my initial psychodynamic training: I think it’s healthy to have losses and do restructuring and arrangements. So I believe it is maturing in many cases and not necessarily harmful.

The concept of development holds together what happens in children and adults, but it seems to limit the pertinence of a unique nosological concept to capture both phenomena.

## Discussion

The first question of our interview grid was always: “*Does childhood depression exist?*” The answer was invariably “*yes*” among our interviewees, often accompanied by an adverb picked in the lexical field of certainty and assertiveness (definitely, of course, obviously, sure). We chose not to put this in the results because we argue that it is no outcome but rather the starting point of our study.

We were cautious not to systematically attribute an ontological meaning to sentences like ‘childhood depression exists.’ In some interviews, it did unambiguously reflect a position rooted in scientific realism. Others were not so easy to map on the realism/antirealism debate. Whether depression exists as an entity of the material world is a continuing and open question for philosophers, to which neither our interviewees nor us can pretend to answer, and certainly not with this study design. Whether there is a phenomenon that our interviewees, the medical institution, and most lay people come to understand as childhood depression is, on the contrary, rather obvious. In that sense, we claim that our interviewees saying ‘yes’ to that first question is not an outcome, nor a metaphysical statement, but the very reason why conducting this study is relevant.

‘Illness’ is the preferred term to describe the subjective and concrete reality of the experience of suffering. ‘Disease’ relates to the abstract medical category, and its status regarding reality is subject to more debate. But doubts about the ontological reality of disease should not obliterate its more tangible social reality. Disease exists inasmuch as the concept produces an effect on society, i.e. what people are willing to do in the name of it (creating institutions, seeking help and care, demanding retribution, defining one’s identity, etc.) [23]. So, ‘definitely,’ ‘of course,’ ‘obviously,’ and ‘for sure’ childhood depression exists in this minimal interpretation. It is precisely because it exists in that social sense, and because one distinctive feature of social reality is that the discourses about this reality shapes it, that child psychiatrists’ understanding of this concept is of interest [23].

### Defining childhood depression

The emergence and critique of the concept of childhood depression were both born in the 1970s. Leon Cytryn and Donald H. McKnew decided to use the term ‘depression’ in 1972 to describe hospitalised children whose mental states appeared, in their opinion, similar to adults with depressive neurosis [24]. They proposed a classification with three subtypes: masked depressive reaction of childhood, acute depressive reaction of childhood, and chronic depressive reaction of childhood.

The link between childhood depression and adult depression was back then rooted in psychoanalytic theory. Although this paradigm is nowadays widely criticised among psychiatrists, three notable features of Cytryn and McKnew’s descriptions seem to live on: the fact that childhood depression is a reaction, that all acute depressive reactions in children are caused by the trauma of loss (death, remarriage, new sibling, moving out, etc.), and that anger is found in all three subtypes. Critiques came shortly after, as Monroe M. Lefkowitz and Nancy Burton wrote in 1978 that “*almost any behavior that is disturbing enough to prod parents into referring a child for professional help may earn for a child a label of depressed*” and that “*if the notions of masked depression and depressive equivalents are considered in the diagnosis, childhood depression reaches a state of omnipresence*” [25] (p. 717).

Of note, when asked about childhood depression, many participants spoke indistinctively of children and adolescents, in opposition to adults. Others spontaneously made a distinction between children and adolescents, but the tipping point was always unclear. When age was brought up, the transition from childhood to adolescence was estimated around 11–13 years old.

The limitation between children and adolescents is blurry because professionals considered them in one continuous dimension: development, at the core of child psychiatry thinking. Child psychiatry is a speciality still searching for its proper place within medicine’s professional and scientific structure. Its tenants are in a delicate equilibrium, having to differentiate it from other medical and nonmedical disciplines and insert it into a common and comprehensive knowledge of mental illness. We contend that the developmental paradigm has a ‘same but different’ explanatory power. It enables the comprehension of a mental disorder as being the same disease across age groups, with a different phenotype following the developmental stage. As such, this paradigm makes rigid age categories irrelevant. Nonetheless, we have seen that it is compatible with both categorical and dimensional approaches to diagnosis. Much like culture in the recent history of psychiatry, it is useful to theorise the unicity of disorders (disease) along with the multiplicity of their expressions (illness). “*Cultural concepts of distress*” appeared in the DSM-5 [12]. Arthur Kleinman wrote in his work on anthropology and psychiatry that “*Depression experienced entirely as low back pain and depression experienced entirely as guilt-ridden existential despair are such substantially different forms of illness behavior with different symptoms, patterns of help-seeking, course and treatment responses that though the disease in each instance may be the same, the illness rather than the disease is the determinant factor*” [26] (p. 450). This analysis, which stems from the variability of depression presentations between Western and Asian adults, could also be meaningful for the variability between children and adults. Participants have said that children have more behavioural and somatic manifestations than adults. The standard clinical picture of this condition may be the unusual one: the cognitive aspects and complex psychology of self-blame and immiserating introspection might be distinctively Western [27], but also adult.

The focus on emotional states versus psychological suffering in the definition of depressive mood amongst our interviewees can be thought through the disease/illness distinction. In a way, emphasising emotions is considering disease as the determining factor, and emphasising subjectively meaningful suffering is considering illness as most relevant. This dispute is not only verbal and conceptual because it has implications for the analysis of scientific data and therapeutic choices. Psychologist Irving Kirsch claims that “*Meanings are not inert*,” that “*they can and do affect people*,” and that “*the way we feel does not depend on the events that happen to us, but rather on the meaning these events have for us*” [28] (p. 136). Theories of meaning can shed light on the significant part the placebo effect is known to play in the treatment of depression. Such statements echo our interviews, for example, with the concept of empathic diagnosis brought on by one participant. Indeed, participants who adhere to the importance of meaningful suffering, independently of the presence of specific affects and emotions, seemed more sceptical of the pharmaceutical efficacy claimed by SSRI adovcates and tended to think that psychotherapy was the only thing that worked (and sometimes, regardless of the psychotherapeutic technique employed).

Why is it so hard to stabilise the concepts of depression and childhood depression? When Participant 5 raised the issue of depression meaning different things for lay people and for professionals (see Results theme #2), she made an interesting point. Depression might be one of those ‘boundary objects’ [29], i.e. concrete or conceptual objects, robust enough to maintain unity but plastic enough to be manipulated in different social worlds. These boundary concepts have the particularity of being weakly structured in the common use but strongly structured in the individual use, much like we have observed in our interviews. In her history of the development of immunology, historian of science Ilana Löwy has shown that boundary concepts have important heuristic roles, and are not necessarily meant to become better defined with time [30]. Loose concepts have cognitive benefits, facilitating the interaction of different scientific cultures to produce new knowledge. They also have social virtues, allowing the development of intergroup alliances and the promotion of particular social interests. They are, therefore, not necessarily a problem and have been shown to be fruitful in scientific processes [31]. Besides, vagueness in words does not imply vagueness in knowledge. Epistemologist Michael Polanyi convincingly argued that we can know more than we can tell, which he called ‘tacit knowing’ [32]. The need for precise criteria to define diagnoses could even put unnecessary intermediaries between clinician and patient, undermining the identification of pathological situations [33]. Perhaps identifying depression or childhood depression is more of a practical knowledge than a theoretical one. Yet, these criteria and these theories exist, are put into words, and have numerous implications.

### Definitional criteria and causal attributions: ethical and epistemological implications

The controversy between using categories and dimensions to define and assess psychiatric disorders is ancient and has been widely discussed by illustrious figures of twentieth century science like Hempel and Eysenck [34, 35]. On the one hand, the categorical approach aims at establishing precise categories, with unambiguously defined features, underpinned by a binary logic: the individual is either subsumed or not subsumed in the category. In medicine, this approach is well exemplified by the creation of syndromes. On the other hand, the dimensional approach considers that healthy and pathological are quantitative differences of the same kind of substrate, only varying in intensity. It leads in psychiatry to the creation of spectrums [36].

This discussion, brought on by some participants, has some practical and ethical implications that are not reducible to the operational goal of classifications. Categories, like the DSM-5’s definition of major depressive disorder, have the disadvantage of producing numerous atypical cases and comorbidities, which is indeed what professionals report in childhood depression. They furthermore create an arbitrary delineation between normal psychology and psychopathology, and can lead to excessive reification of diagnoses, i.e. the belief that these categories are a pure mirror of nature [36]. On the contrary, dimensional approaches facilitate diagnosis from nonspecific symptoms, avoid premature conclusions from fully syndromic approaches, and allow for a better translation of therapeutic efficacy in clinical trials [36]. Therefore, it is not surprising that several participants have advocated using dimensions over categories in childhood depression. However, dimensions have some downsides which should not be overlooked. They weaken the communication between professionals (reliability), which was at the core of the DSM-3 project. Perhaps more worrying, they also break down the barriers between normal and pathological, by conceiving them both on a single continuous axis, which could lead to a massive utilisation of psychotropic medicines [36]. In the end, a categorical approach suggests that the problem is something that the patient has, whereas a dimensional one suggests that the problem is something that the patient is [37]. This issue is far from being ethically neutral, and the debate between categories and dimensions should not be simplified to purely technical and theoretical questions. Besides, medicine’s pragmatic goal—diagnosis for treatment, not for knowledge—generally leads to set cutoffs between normal and pathological along continuous dimensions, reverting to a categorical reasoning [37]. There are indeed very different kinds of categories: all do not need to fit nature’s underlying reality adequately, but all have a precious practical utility in action-oriented endeavours like providing health care [38].

The definition of depression in relation to its cause is also subject to many debates with many implications. Sociological studies describe a drift in psychiatry’s causation model from personal and interpersonal individual history to biological mechanisms [39]. While this analysis may be accurate in the production of knowledge, we cannot say the same about our interviews with clinicians. As we have seen, the participants kept claiming to look for causes of childhood depression in their life history. Among our interviewees, including the minority who rejected genetics as being epistemologically relevant to childhood depression (which we should not overgeneralise to child psychiatry as a whole), the dominant paradigm was a biopsychosocial model [40], not a biological one. The biopsychosocial model is exemplified for mental disorders in the ‘stress-diathesis’ model: genetic influences combined with unshared environmental influences make the baseline biological susceptibility to psychiatric illness, and later life events are triggers for the occurrence of mood episodes. As psychiatrist Nassir Ghaemi puts it, “*later life events do not cause the illness in a general sense; they only determine how frequently and when the illness reveals itself in mood or psychotic episodes. The episodes are part of the illness, not the entire illness. […] Both life events and underlying biological susceptibility are legitimate targets of interventions, with psychotherapies and medications, respectively*” [41] (p. 201). But he does not oppose biopsychosocial to psychosocial and/or biomedical; he claims that the biopsychosocial model is a “*vague eclecticism*” that is as dogmatic as the psychosocial and the biomedical alone, leading to give everyone everything, irrespective of what works best. It is falsely pluralist in that it mixes various scientific methods and theories of mental health without working on which is the better, problem by problem [41].

Which model would then work best for childhood depression? Firstly, there are ethical considerations to take into account. We had anticipated that different theories on childhood depression would lead to different therapeutic strategies. Yet, these strategies were surprisingly homogenous despite the epistemological dispute on whether environment or biology was the leading cause. Therefore, we tried to look for other motivations to adhere to one or another epistemological model. A biomedically dominant model of disease has great destigmatising virtues, as exemplified by Participant 3’s caution to not blame parents (see Results theme #3). Biological explanations of disease allow to take off stigma attached to psychosocial explanations, which may partially explain why they are largely advocated by patients, loved ones, and associations [39]. Psychologist Ilana Singh showed in a study on ADHD that parents tended to say their child did not have a behavioural problem, but their brain did; thus considering they knew the true and authentic child while ADHD was alien and inauthentic [42]. On the other hand, psychosocial explanations do seem to spark a much welcome change in the child’s social environment. Parents, in particular, were in this perspective strongly prompted to change their attitude. The label of ‘depressed’ itself could be problematic for these psychiatrists if understood in a biomedical way. For example, Participant 12’s ‘empathic diagnosis’ fits this approach.

Choosing between a biomedical and a psychosocial model for childhood depression also has deep epistemological implications. All the participants agreed to say that psychosocial causes were more influential in childhood depression compared to adults. In a way, it means that childhood depression is a sensible human emotional response to adverse life events. ‘Sensible’ here is used in the literal sense ‘that we can make sense of’ and does not mean ‘okay’ or ‘unproblematic’, because there is no denying the terrible social, developmental and psychological consequences for these children. ‘Sensible’ means that there is a socially understandable reason for such an emotional response. Sociologists Allan V. Horwitz and Jerome C. Wakefield have suggested that distinguishing normal sadness from pathological sadness could only be done by assessing the causes, not the state of sadness itself [4]. They contend that this has been crucial throughout the history of psychiatry, for Hippocrates, Freud and even Kraepelin, to whom it is customary to attribute the birth of a biomedical model of psychiatry. Indeed, even after the nosographic twist of the DSM-3, the diagnosis of MDD was subject to the ‘bereavement exclusion’: i.e. that depression could not be diagnosed with the same criteria after the loss of a loved one. It seemed reasonable; it also seems likely that other life events could explain episodes of intense sadness. Yet, in 2013, the bereavement exclusion was removed from the DSM-5, precisely because no differences could be found between psychological states of sadness in bereavement and MDD [43]. This move enshrined the end of psychiatry’s ‘causeless tradition’ in the diagnosis of depression.

The extreme distress that anxiety and sadness can cause is not to be taken lightly and undoubtedly call for care, but if we consider it part of ‘normal’ human functioning, it does, however, raise the question of the medicalisation of such suffering. All our participants advocated for a multidisciplinary care of depressed children to try and activate all levers: psychotherapy, medication and social life. If medication is in most cases useless and avoidable, if physicians do not perform psychotherapy and social care (because they are not trained for it, because it is out of their prerogatives, or simply because they do not have enough time), and if the pathological status of childhood depression is doubtful, is there still a rightful role for medicine to play?

### Medicalisation

There is no moral overtone in our use of the word ‘Medicalisation.’ It is a mere description, not value laden like ‘overmedicalisation’. Over the last couple of centuries, there has been a general tendency to increase medicalisation of social behaviours, albeit paralleled with demedicalisation of certain behaviours (homosexuality, masturbation) [44]. The concept of childhood depression in its current use can be thought through this larger medicalisation process. Yet if medicalising nonpathological states should be questioned, it cannot be an argument per se. Birth pain is considered normal, and this does not automatically lead to saying the involvement of physicians should be stopped because pregnancy is not a disease.

The case of ADHD is an interesting example of the medicalisation process. Initially, only hyperactive children were targeted. Attention was then added to the syndrome and eventually broadened to adults in the 1990s, leading to a medicalisation of underperformance [44]. Perhaps depression, first losing the imperative of causelessness, then being adapted from adults to children and added other symptomatic behaviours like irritability, is undergoing a similar process.

“*Persons are in the psychiatric realm when their body, psychology and/or particular expressions of emotional and mental distress are constructed as ‘mental illness’ or ‘disorder’*” [45] (p. 1182). For Horwitz and Wakefield, “*All professions strive to broaden the realm of phenomena subject to their control, and whenever the label of disease is attached to a condition, the medical profession has the primary claim to jurisdiction over it*” [4] (p. 213). For them, the medicalisation process is promoted by the profession of medicine, as doctors are the main beneficiary of a definition that allows labelling and treating previously nonmedical problems as disorders. Conrad and Slodden make a different analysis: clinicians are not (anymore) the major drivers of this medicalisation process. As we have seen, all our participants were likely to adopt an eclectic biopsychosocial model, and to downplay their part in the care of psychologically suffering children in favour of a wide network of medical and nonmedical care. In a qualitative study with 60 participants (depressive adults and their doctors), Kokanovic, Bendelow, and Philip made similar observations [21]. They found that physicians easily took liberties from the DSM categories to diagnose depression and that they experienced a tension between the biomedical discourse of depression in which they were trained and which accords them clinical authority, and the recognition that the social context of patients’ lives contributed to their experience of emotional distress. As a result, many prescribed antidepressants reluctantly. On the patients’ side, although they agreed that the source of their suffering was outside of medicine’s scope (life events), their first instinct was still to consult a doctor, short of any other accessible kind of help and care. Since the 1980s (at least in Western countries), promoters of medicalisation have instead been medical care consumers, insurances, and the pharmaceutical industry, with doctors increasingly becoming gatekeepers rather than advocates [44].

What are the consequences of such medicalisation? This is a subject for another study altogether. We can however make some remarks based on our material and the literature. The participants said that some of their patients were in extremely severe condition, with high risk of dramatic social consequences or even death, prompting them to give medication even when they were not convinced it was useful. There is probably a selection bias towards most severe cases in our sample of recognised international experts, often working in tertiary and quaternary health-care facilities. No doubt that the amount of urgency and risk associated with these cases called for radical interventions—radicality within the power of the medical institution. Given how problematic they are, such cases probably do not allow to explore the frontier between normal and pathological. Therefore, using this minority as a starting point to define the whole concept of childhood depression is likely to play a role in pathologising what would otherwise be normal sadness and irritability in less severe cases. For the majority, the use of medication (which is more personality enhancers than antidepressants according to many researchers [4, 28, 41]) is far from being insignificant. The side effects are not negligible and the benefit/risk equilibrium could be revised. On the individual level, it does raise the question of the (yet unknown) physiological role of sadness and anger, and potential harm of blocking their manifestations. On the collective level, it erroneously misconstrues social problems as personal problems. Indeed, with the way we frame the concept right now (both in children and adults), it is not surprising that epidemiological studies find enormous rates of prevalence (since it will pick up states of lesser emotional distress that will not necessarily lead people to consult a physician) and qualify depression as a major public health concern [1, 4]. It does catch up with Lefkowitz and Burton’s 1978 concern that depression could reach the state of omnipresence.

### Strengths and limitations

Overall, our study meets a fair amount of quality criteria for qualitative research [46]. Credibility is ensured by the prolonged engagement of two researchers full-time for six months each, peer debriefing within the larger research unit and with an external group of researchers, and a large portion of our data is provided in the Supplementary materials. The detailed account of where the participant population came from and why they were recruited ensures good transferability. Dependability and confirmability were assessed by triangulation to ensure a satisfying fit to the data, the avoidance of the inquirer’s terminology and a priori concepts, and surveillance of potential methodological shifts. Trustworthiness might have been further improved by the use of a full audit or a reflexive journal.

There are some limitations in our study. Intrinsic to the methodology of interviews is the potential gap that could exist between discourses and actual practices. This is why we have focused so much on the concepts and the way professionals arrange them, than on the phenomena themselves. Ethnographic observations should be envisaged in future research, to measure the importance of this gap. Likewise, to frame the discussion more on actual phenomena and less on concepts, interviews which children and their families should be considered.

Some limitations are also strengths in our opinion. First, having conducted the analysis by researchers working on transcripts produced by other researchers increases the risk of distortion in the interpretation of participants’ discourses. However, involving several researchers is a more robust process, as science is by definition an intersubjective and collective activity.

Second, interviews were conducted in two different languages (French and English). Analysing discourses is thus harder because it gives a lot of importance to the words themselves. Furthermore, many participants were not native French or English speakers, and sometimes their fluency was an obstacle. The importance to give to the choice of certain words and certain syntaxes was sometimes difficult because it was unclear whether they were chosen on purpose, or short of better language skills. Nonetheless, having participants coming from all six continents was extremely precious and a rare feature of qualitative studies. The language barrier is probably an acceptable limitation in contrast to the strength of an international design.

Finally, we have not seen as many culturally linked views of childhood depression as we had anticipated. It may mean that the concept is not as culture relative as we think. But as recognised figures and reference professionals worldwide, it is also likely that their affiliation to this common international community homogenise their views on the question. Yet, as major contributors to the intellectual livelihood of the field, their reflections are also more likely to impact the medical community as a whole and the construction of knowledge.

## Conclusion

Using a constructivist approach and a qualitative study design, we have been able to explore how early twenty-first century international experts in child psychiatry shape the concept of childhood depression. Most participants agreed that childhood depression was a mental disorder in which irritability was prevailing, heavily influenced by psychosocial factors, and for which psychotherapy was the ideal treatment. Many points of subtle dissent have also surfaced: whether depression is primarily a mood or a psychological mechanism, whether categories or dimensions are more suitable to make the diagnosis, and whether there is a genetic predisposition were some of the most controversial topics.

Child psychiatry is increasingly gaining acceptance and independence in medical institutions across the world. Like its adult counterpart, its body of knowledge and theories is a constant work in progress, subject to displacements and refining. Definitional criteria and causal attributions are not mere verbal disputes, they have concrete epistemological (what is normal, what is pathological?), ethical (who or what gets blamed for causing the disease?), social (what is medicine’s role?), and political implications (what is of major public health concern?). These theoretical discussions should not be overlooked and must be continued in further research.

### Supplementary Information

Below is the link to the electronic supplementary material.

Supplementary file1 (DOCX 32 KB)

## Data Availability

The original data for our study cannot be shared openly to protect study participant privacy. We however provide in a supplementary file a large portion of (anonymised) quotes drawn from the raw material, which were are the foundation of our analytical work.
