# Supplementary material for: Constructing childhood depression: a qualitative study with international experts in child psychiatry
Source: Eur Child Adolesc Psychiatry. 2023 Aug 30;33(6):1847–62. doi: 10.1007/s00787-023-02270-0 (PMC11211152; doi:10.1007/s00787-023-02270-0)
Supplement: Supplementary file 1 — Supplementary file1 (DOCX 32 KB) [file 787_2023_2270_MOESM1_ESM.docx]

Constructing Childhood Depression

# Supplementary Material

## Semi-structured interview guide

**STEP 1 : CLINICIAN WELCOME**

- Request for authorization to record, reminder of the challenges of the interview, the conditions of its conduct, the topic addressed and the anonymous nature

**STEP 2-TIME OF QUESTIONING AND EXCHANGES**

**-**introduce yourself ( job, place , age)

**-**childhood depression : what is it for you? Does it exist? Is it different from adolescent’s depression ? from adult’s depression ?

**-**what do you think about DSM criteria ?

**-**how do you recognize childhood depressive disorder ? what are the clinical signs ?

**-**are somatic complains as depressive equivalents ?

- do you use ay rating scales or tools ?

-few authors speak about child suicide in the literature, have you already seen suicidal child ?

-which treatment do you consider preferably? Which kind of psychotherapy ? which king of antidepressants ? what do you think about fluoxetine ?

-do you identify any risk factors ?

-do you identify any comorbidities?

-what do you think about genetic predisposition?

- Is there a relationship between child depressive disorders and then psychopathology of adolescent?  Do depressive disorders lead to vulnerability to adult age ?

**STEP 3- CONCLUSION**

- Talk to the clinician (Does he have any comments to add?)

- Acknowledgments and asks if clinician would like to be included in study acknowledgments

## Quotes

| **What Makes ‘Childhood Depression’ Depression** | *Mood, affect, emotion* | Yes, a child will be abused. Yes, a child will not do well. Yes, they will be agitated instead of being sad. All that is true. But is it depression? (P1*)  DSM-5 concerns adult depression. I think they are not the same. I think childhood depression should be studied at its own credit and merit. (P6)  They might be very very depressed and the next minute we might get them involved in doing something and then they turn back to being depressed. They are very ‘here and now’ creatures. (P6)  To me, it is clearly a separate entity. (P8*)  When I witness an obvious case of sadness with some sort of psychomotor retardation and suicidal ideation, I am suspicious. […] There is something wrong, it is too good to be true. […] It is a child who wears one of their parents’ depression symptoms, [or] it’s a child who borrows from the adult depressive repertoire, but it’s often inauthentic in the sense that it is not the child’s issue at the forefront. […] I am against diagnosing a child that easily, with a major depressive presentation based on an adultomorphic clinical picture. (P15*)  There isn’t the classic depressive affect that we can see in adolescents and adults. (P18*) |
| --- | --- | --- |
|  | *Psychological suffering* | Let’s think of this more broadly than just as a disease condition, let’s think of this as a feature of the way life seems to this person right now. (P12)  We are very compliant and lawful regarding the obligation to make a diagnosis based on an existing classification and a well-defined practice: we call it the clinical discussion. Then the psychopathological discussion: now, we are going to understand the child, from their history and from what has happened in their life. And at that moment, yes, depression becomes meaningful. (P15*)  MDC: Is it different from adolescent and adult depression?  P5: Yes because children usually cannot verbalise themselves so the adults around them see changes in their functioning.  I am not sure about the reason for the difference but I suspect it is different because of differences in social and cognitive maturity in adolescents, but differences in brain maturity and biology may be different, as well. (P3)  MDC: Is it different from adolescent or adult depression?  P6: I think it is because children are different and maybe they are unable also to formulate it the way that adults or adolescents will formulate it but this nonetheless makes them not less vulnerable to depression. […] It’s the same feeling, it’s an affective disorder, it’s about the mood being low, I mean this is in common with all age range but the how of expressions might be different than in adolescents and others.  It’s a mood disorder and there are some differences because it depends on the age of the child: they cannot express in words what they are living or what they are feeling so maybe, it is more of a behaviour thing than in other ages. (P13)  Childhood depression is different from adolescent because it is often expressed through the body, and oftentimes the child does not know how to describe their emotions and feelings well. By the way, the classic concept of ‘masked depression’ acquires its full practical significance here. (P15*)  I would rather set the bar at 12-13 years old with indeed an ability to verbalise depressive affects that does not exist so much younger. (P18*) |
| **How To Make a Diagnosis in Child Psychiatry** | *Category, dimension, and development* | We consider depression not as a categorical condition but a dimensional condition. I think we see all the children’s disorders in a dimensional way. (P4)  I think the DSM is too narrow [...], it puts too much emphasis on the categorical diagnosis; we use these terms, categorical versus dimensional diagnosis. (P12)  Childhood depression would be, in my opinion, more dimensional, let’s say, than a clear-cut nosographic picture like we see in adults. (P15*)  DSM is useful, it’s a heuristic and it’s a guide, but it shouldn’t be reified. (P10)  I think that they’re not as developmentally sensitive as they might be. […] I think what DSM-5 said is ‘this is what it looks like in adulthood; let’s now look back and see what it might’ve looked like in childhood’. I just think they have it backwards. (P3)  The how of expression of the child must be taken in his developmental stage. (P6)  You have to admit that the criteria are beginning to show some developmental differentiation. […] The risk is that one might use the adult criteria without knowing these fundamental developmental notions. (P16*) |
|  | *Making sense of a category: the time factor* | Especially, the change in the amount of activity is helpful. (P7)  You have to look for change compared to the previous state because there are children who have always had… Parents tell me ‘he has always had this look, he has never been very social, he has never been a funny kid’. (P8*)  In fact, in childhood depression, there has to be a rupture with the previous state. […] The time factor of the clinical work is very difficult to incorporate in the DSM. (P9*)  So, my guiding principles, to answer your question, are: (1) clear-cut change; (2) interviews with the family and a clinical examination replicating the classic symptoms of depression, that is, manifestations of a change in the cognitive sphere, a sharp decline in school performances. (P15*)  MDC*: From a semiological perspective, what do you observe in children who have depression?  P16*: I think, for starters, that it is self-esteem and the notion of change. That is something we see, a change in the functioning.  When a mother tells me “they used to be a very joyful child, always on board to do anything, but something has changed, they don’t have the same livelihood, the same enthusiasm for everything”. (P18*)  *Parents’ role in assessing the time factor*  First of all, I think you have to trust parents when they come in and they say ‘there’s something wrong with my child. […] I think that’s the starting point. Because parents are, for the post part, very good observers of their children. (P3)  You know, parents would say in [the local language] ‘the bubble is gone’, their eyes are dim. So I have not yet seen a system which is putting this phrase into consideration but it’s true that parents would note that the child is no longer as bubbly and lively as they used to be. (P6) |
| **Why Childhood Depression Happens** | *Environment: culture, society, and life Events* | *Structural factors: society, economy, culture*  With these children, there is a chronic problem coming from the—we say ‘physical’, although I don’t like separating [from mental]—disease, and it is always pretty tough for families. It’s a public hospital, they are poor people with a long-term [comorbid] medical problem, and these people have a lot of difficulties going through this. So depression is almost a logical consequence. (P2*)  I think profound deprivation. What do I mean by profound deprivation? Not necessarily orphanages, but I think poverty, and social isolation, inadequate diet and poor access to clean water, and less schools. I think that those kinds of environmental factors are profoundly demoralising. (P3)  Also, there are some socioeconomic factors. It’s more frequent in the low socioeconomic level. (P13)  *North-African specificities (no participant number to preserve anonymity)*  An important risk factor: you have school pressure, like I told you, a sort of school terrorism exerted on children.*  When they don’t have any self-confidence, when they are a little shy and have bullying problems at school, etc., completing several stages of the game [the blue whale challenge] until suicide can be heroic and give them back some confidence. There are many children who committed suicide in Morocco.*  To add to the cultural dimension, it is a country were school pressure is tremendous. We have two periods for paediatric suicide attempts: the December/January period and the June period, which correspond to mid-term and final exams and results. So children are under so much academic pressure that when it doesn’t work for them, many are tempted by suicide attempts.*  Death can be a solution. It is sort of glamourised in this very peculiar economic context. I don’t know if you are acquainted with Tunisia, but we have been since 2011 in an era we qualify as ‘post-revolutionary’. It started with someone who self-immolated. Many adults still do… have done it, thankfully less now. But every time there is a problem, every time someone sees on obstacle along the way, they threaten to set themselves on fire in the street. We have seen a lot of this.*  *Significant life traumas*  Is cause the prime factor [of definitional criteria]? That they were abused, then they had a biological stress, so psychology of biology. And it translates into symptoms we would call depression. (P1*)  All started before 23 or 24, so it is the same, but we have the advantage to be at the beginning, so i think is very different if we treat these depressed kids in the first period that they have depression or the anxiety or if nobody supports them and then you are 37 and have a change in your job or in your family members or whatever or you have a tragic situation and you are traumatized and depressed and you say I had no idea. (P4)  The youngest children was at the age of 7 who attempted a serious suicide attempt. She jumped out of the window from a high building and she broke a leg. She had trauma difficulties and was recently placed in a foster family because of the severe neglect and abuse in her primary family, so that was a reaction to her separation from her parents. Well, see, these in children is not as common as in adolescence but sometimes it happens. Some of the children have really severe depression that was not recognized. (P5)  It can start with the birth trauma even, so we are aware that even very very young children can be very depressed. (P6)  MDC*: In your practice, have you identified any risk factors? P8*: Parents who are either totally negligent, or put too much pressure, are never satisfied, who are violent; history of sexual abuse or unwanted sexual touching are very frequent and it takes time to bring it to light.  MDC*: Do you identify any risk factors? P13: Yes, traumatic events and maltreatment, sexual abuse, physical abuse, emotional abuse and also neglection.  When it occurs in the young childhood in particular, we’re always concerned about potential factors in the environment that could have caused it, so things like abuse. (P14)  MDC*: What have you been observing in terms of risk factors? P16*: We have a lot of life events, like abuse, or less dramatic experiences but still painful and hard.  Of course life events, we all know, would have an important role to play. More and more we are seeing adverse childhood experiences, childhood trauma, traumatic events for attachment relationships, current relational issues, break-up in relationships whether it’s family relationships or whether it’s, you know, boyfriend-girlfriend relationships: any break or loss of any kind would all be considered as factors. (P17)  *Social impact of other disorders*  What we do have is a combination of things, so we have plenty of children that have inside disorders and depressive features, ADHD and depressive features or some conduct disorders that have some depressive features, but it’s very rare that we get just a depressed case. (P4)  It’s always a consequence of the main disease, diabetes in this group of patients. That’s why for me, it’s always hard to see ‘simple’ cases. It’s not exactly depression coming in, it’s almost always a consequence of the disease, of other comorbidities. (P2*)  When you talk to the child and through the overall assessment, then you see that the child is not like depressed but the family interactions that the parents don’t spend enough time with them. […] Children from age 10 to 12 come and say ‘I’m depressed’ but don’t have emotional support in their families, it’s not like a real depression. (P5)  It is definitely a comorbid disorder and the other disorder is the major disorder. So obviously, major depression is a common comorbid disorder. (P11)  It’s very difficult because many patients of PTSD have the experience of child abuse. […] It is often difficult to give a differential diagnosis between depression and PTSD. (P7)  *Somatic complaints as anxiety equivalents*  Somatic symptoms indicate where there is anxiety because in children, most of the time, they have anxio-depressive disorders; pure depressives, without anxiety, are exceptional. (P9*)  The somatic complaints, I wouldn’t immediately associate them with childhood depression. […] I see them more in relationship to anxiety disorders. Obviously, anxiety disorders can also be associated with, so it could be comorbid depression. But the somatic complaints would most likely tend to be associated more with the anxiety disorder than the depression itself. (P11)  MDC: A few colleagues speak about somatic complaints like depressive equivalents? What do you thing about this? P13: Especially when there is a comorbidity with anxiety, I think that these symptoms are important.  Here, the body is at the forefront, we will have a lot more somatic manifestations of anxiety. (P15*)  *Anxiety and ADHD*  Not so many difficulties in diagnosing them as depressive disorder, but many patients are misdiagnosed as an anxiety disorder. (P7)  Anxiety of course is a precursor in a way. You want to think about it for some children, that, if you don’t treat the anxiety, over time they become depressed. (P14)  The severity of anxiety leads to depression. The history and length of anxiety disorders too lead to depression, maybe through exhaustion. (P18*)  Many children have these acting out reactions because of the irritability and we have to make assessment whether it’s a behavioural disorder or depression. But children with depression usually feel guilty when they act out, when they act aggressively. So that’s how we distinguish depression from ADHD. (P5) |
|  | *Biology: brains and genes* | MDC: Do you think there is a genetic predisposition?  P6: I think so, but it doesn’t mean that they have to have it”.  Depression, like money, can run in families. However what I try and communicate is that the genetics is not your destiny, you can certainly pay attention to the psychosocial supports in your environment. […] Genetics is a small component, the bigger one is the psychological factors. (P10)  MDC: Do you think there is a genetic predisposition?  P12: Yes, I think there is, I don’t think it accounts for everything in depression but it certainly is part of the story.  Childhood depression could have a genetic component if for example the mother is depressed, but the mother’s depression may lead the child to be depressed due to environmental factors. (P14)  It goes without saying that the biological determination is a risk factor, of course life events we all know would have an important role to play. (P17)  There are environmental factors that play a role in mood disorders and the answer is I suspect that they may not account for a huge portion of the aetiology, but they certainly can count for a huge portion of the aggravating factor. (P3)  This makes me really think a lot of what exactly depression is, and maybe there are other neuropeptides or whatever. We just don’t have the slightest clue what to do with these kids other than psychotherapy. […] I think it’s only effective in kids with low leptin levels, I don’t think it’s a general treatment for depression. (P11)  I have no idea about genetic predisposition. (P7)  MDC: Some colleagues speak of genetic predispositions. What do you think about that?  P9: I don’t believe it one bit. I find it totally absurd. Data do absolutely not go this way. […] We are just saying that psychiatric pathologies have something to do with brain development and this, we already knew. So I am very cautious.*  I only settle for the predisposition to bipolar disorder. (P15)  MDC: Some colleagues speak of genetic predisposition, what do you think about that?  P18: I think, in my opinion, that we don’t have enough evidence for it.  *Perception of parents by clinicians according to disease model*  It’s not parents who cause it. And I think we need to stay away from, when we do diagnoses and we talk about treatments, we need to be very careful to not blame anybody, because I think that’s just not very helpful in the process of treatment and recovery. And if it were parents who caused it, when the kids grow up and then they have recurrent depressions, I think it’s unlikely that when you’re 35 years old, that gets your mother’s fault that you have depression, I mean, it just seems unlikely. I think we need to be careful about that along the way. (P3)  [I say to the parents] “they’re hurt, look, it’s normal! They hear you yell at each other, they sometimes hear shouting and are afraid for themselves”. [...] Just saying “stop messing around” [to the parents,] they understand 3 out of 4 times. (P1*)  It’s more, I think, incompetence on behalf of the parents, not knowing what to do in particular circumstances, not being able to enforce stuff at home. So these kids have a lot of power and then proceed or use this to, I don’t know, get attention. (P11)  I won’t use the word ‘depressive’ because when I say ‘depressed’, for them someone ‘depressed’, either they don’t know what it is, or it’s someone knackered and tearful. They’re not crying so the word ‘depressed’ is useless. I am going to say “so here is the thing: they’re in pain, and their way of dealing with the pain is to hit schoolmates and lash out at their teacher. (P1*) |
| **How To Deal With Childhood Depression** | *Medication* | If after normally 4 to 6 weeks of treatment we don’t see change, we don’t see progress, then we add medication. […] If it is ADHD, we don’t need the fluoxetine, we use it for depression. (P4)  After three or four weeks, we would start with an antidepressant. For children, basically, almost always fluoxetine (P11).  For all the depression disorders, the important is psychotherapy, and this psychotherapy must involve the family and also the school in most of the times. But for severe, it’s also important to include antidepressants. (P13)  MDC*: Recommendations speak of fluoxetine above 8 years old. Below 8, do you sometimes prescribe? P8*: Never. I think there is not enough evidence or clinical trials. It is still a developing brain.  Some parents get very alarmed because the child feels very tired and drowsy because whenever they are given psychotropic medicine, at least in [country], they think it’s working on the brain so the brain is very important for studying. (P14)  There are many cultural things in this. What would a family [of my nationality] want is categorically refused by a family in [my country of practice]. Actually, and in contrast, families [here] want the pharmaceutical treatment because they consider that depriving the child of treatment is to make them suffer for no reason, and to waste their precious development time. Whereas in [my country of origin], it is the opposite. [There], we consider that we have to avoid medication at any cost, that it is toxic for the development of the nervous system and delay and avoid as much as possible. So, in my practice, I do like [people here] because I am [here]. (P18)  Therapeutic trials of depression in children were made from criteria which are adult criteria. […] And randomized trials in children need the approval of the parents to include them, need the phenotypes to be homogenous and well known. But we don’t: we make it up from adults, so the data is very poor. (P1)  The patients of childhood depression often do not respond to drug therapy. (P7)  Actually, when you look at the response to pharmaceutical treatment in children, you realise that antidepressants do not work. Potentially, fluoxetine has a small efficacy, but a really tiny one. (P9) |
|  | *Psychotherapy* | I give priority to good psychotherapists. That’s what works. There is even data about it. (P1*)  When a child comes see a shrink, it’s not because the professional is labelled ‘psychoanalyst’ or ‘behaviourist’ that it’s in any way meaningful to them. The child comes to see an adult who cares about them, who asks them questions, who wants to help them. All that produces a psychotherapeutic process. […] In the end, even an ignorant can help a depressed child. […] In fact, when you look at the detail, all psychotherapies work in depression and now, there are even large-scale studies. (P9*)  In practice, things are different. It’s great to say that CBT is the first-line of treatment. But then, when you have a year and a half waiting list like [here] in every department now, you can’t do that. So we are talking of ideal, complex ways to do. But eventually, unfortunately, most cases do not have immediate access to the type of psychological therapies we want. So the question is: should we initiate medication even if the guidelines clearly say that medication should be used in combination with psychological treatments? […] So there it is, I would like to do things differently. But unfortunately, practical constraints lead us to do otherwise. (P16*) |
|  | *Social care* | We remove the kids from home for one, which is a major intervention. Then we provide the kids with a daily structure which they commonly did not have prior to coming into the unit. (P11)  We also work with the parents, then we inform social care to be involved in the family. (P5)  When choosing treatment, I mainly adjust environment for childhood depression. (P7) |
| **Child Psychiatry: A Rising Speciality** | *A speciality in its early institutionalisation* | When I started being a psychiatrist, most of my colleagues that were not psychiatrists said ‘what do you do, you attend crazy kids? And when we went to the school of medicine, we only had one class of child psychiatry. (P13)  We have just started, two years ago, a training programme for residents. We now train our own child psychiatrists [here], which is new and gives us a lot of hope to develop the discipline further in years to come. I am in charge of this. Child psychiatry becomes for residents a speciality in its own right, ‘independent’ if I may say so, which means we don’t depend on adult psychiatry to train residents anymore. At the university level though, it is still the big psychiatry department with a research chair in child psychiatry. (P18*) |
|  | *Child psychiatry’s scientific paradigm: development* | I think we have to push more as an academic child psychiatry unit to really show the differences relating to development. (P16*)  There is an idea of continuity. I think so myself. In my opinion, indeed, a child depressed since childhood is a lot more at risk to be a depressed adolescent and a depressed adult. (P2*)  I definitely think that there is major depressive disorder in children. It is often antecedent to major depressive disorder in adolescents. (P3)  If they live in a dysfunctional environment, they’re harder to treat and they might continue to have problems in the adult age. (P5)  Yeah, there is a continuation particularly if the psychosocial situation are continuous, as parental discord or abusive family or domestic violence. If the psychopathology of the environment continues, I think you don’t have much chance not to continue with your own psychopathology. (P6)  Maybe we should be careful with that [continuity], because there are children who have lived difficult things that make them stronger afterwards. Many papers say that. We build our lives with the obstacles we have encountered. The risk with saying “oh yeah, they’re depressed, they’ll keep on being depressed” is that it becomes self-fulfilling. (P1*)  A significant group will have an episode of depression but they’ll either get some significant changes in their environment and make better choices, or they’ve learned something and they do well, they may get episodes of dysthymia and some anxiety but they learn a way of coping with it and they don’t have to come back for mental health services as an adult. (P10)  MDC*: Do you think that depression in a child can lead to a psychological vulnerability at adult age?  P15*: Not particularly. I even think it can be maturing. I here go back to my initial psychodynamic training: I think it’s healthy to have losses and do restructuring and arrangements. So I believe it is maturing in many cases and not necessarily harmful. |
